# Associations of *pri-miR-34b/c* and *pre*-*miR-196a2* Polymorphisms and Their Multiplicative Interactions with Hepatitis B Virus Mutations with Hepatocellular Carcinoma Risk

**DOI:** 10.1371/journal.pone.0058564

**Published:** 2013-03-13

**Authors:** Yifang Han, Rui Pu, Xue Han, Jun Zhao, Yuwei Zhang, Qi Zhang, Jianhua Yin, Jiaxin Xie, Qiuxia Shen, Yang Deng, Yibo Ding, Weiping Li, Juhong Li, Hongwei Zhang, Guangwen Cao

**Affiliations:** 1 Department of Epidemiology, Second Military Medical University, Shanghai, China; 2 Division of Chronic Diseases, Center for Disease Control and Prevention of Yangpu District, Shanghai, China; 3 Department of Hepatobiliary Surgery, Eastern Hepatobiliary Surgery Hospital, Second Military Medical University, Shanghai, China; 4 Department of Ultrasonography, Changhai Hospital, Second Military Medical University, Shanghai, China; 5 Health Examination Center, Changhai Hospital, Second Military Medical University, Shanghai, China; Drexel University College of Medicine, United States of America

## Abstract

**Background:**

Genetic polymorphisms *of pri-miR-34b/c* and *pre-miR-196a2* have been reported to be associated with the susceptibility to cancers. However, the effect of these polymorphisms and their interactions with hepatitis B virus (HBV) mutations on the development of hepatocellular carcinoma (HCC) remains largely unknown. We hypothesized that these polymorphisms might interact with the HBV mutations and play a role in hepatocarcinogenesis.

**Methods:**

*Pri-miR-34b/c* rs4938723 (T>C) and *pre-miR-196a2* rs11614913 (T>C) were genotyped in 3,325 subjects including 1,021 HBV-HCC patients using quantitative PCR. HBV mutations were determined by direct sequencing. Contributions of the polymorphisms and their multiplicative interactions with gender or HCC-related HBV mutations to HCC risk were assessed using multivariate regression analyses.

**Results:**

rs4938723 CC genotype was significantly associated with HCC risk compared to HBV natural clearance subjects, adjusted for age and gender (adjusted odds ratio [AOR] = 2.01, 95% confidence interval [CI] = 1.16–3.49). rs4938723 variant genotypes in dominant model significantly increased HCC risk in women, compared to female healthy controls (AOR = 1.85, 95% CI = 1.20–2.84) or female HCC-free subjects (AOR = 1.62, 95% CI = 1.14–2.31). rs4938723 CC genotype and rs11614913 TC genotype were significantly associated with increased frequencies of the HCC-related HBV mutations T1674C/G and G1896A, respectively. rs11614913 was not significantly associated with HCC risk, but its CC genotype significantly enhanced the effect of rs4938723 in women. In multivariate regression analyses, rs4938723 in dominant model increased HCC risk (AOR = 1.62, 95% CI = 1.05–2.49), whereas its multiplicative interaction with C1730G, a HBV mutation inversely associated with HCC risk, reduced HCC risk (AOR = 0.34, 95% CI = 0.15–0.81); rs11614913 strengthened the G1896A effect but attenuated the A3120G/T effect on HCC risk.

**Conclusions:**

rs4938723 might be a genetic risk factor of HCC but its effect on HCC is significantly affected by the HBV mutations. rs11614913 might not be a HCC susceptible factor but it might affect the effects of the HBV mutations or rs4938723 on HCC risk.

## Introduction

Persistent infection with hepatitis B virus (HBV) and/or hepatitis C virus (HCV) is the major cause of hepatocellular carcinoma (HCC) worldwide. Most HCC cases occur in Eastern and South-Eastern Asia and Middle and Western Africa where chronic infection with HBV is endemic [1]. The overall sex ratio (men: women) of HCC incidence is 2.4∶1, while in the population seropositive for hepatitis B surface antigen (HBsAg) only, the sex ratio is 3.4∶1 [2], [3]. High HBV replication status, active and persistent inflammation in liver, HBV genotype C (*vs.* genotype B) and viral mutations in the enhancer II/basal core promoter/precore (EnhII/BCP/PC) and the preS regions of HBV are significantly associated with increased risks of HBV-related HCC (HBV-HCC) [4]–[8]. Interactions of viral and genetic factors are believed to influence the occurrence of HBV-HCC. Recent genome-wide association studies have identified that single nucleotide polymorphisms (SNPs) at loci encoding proteins related to the immune and inflammatory responses are associated with HBV-HCC [9]–[12]. Pathway-based approaches have also determined that SNPs of key inflammation-related molecules including NF-κB and tumor suppressors such as p53 are closely associated with HBV-HCC [13], [14]. Genetic susceptible factors might predispose the HBV-infected subjects to the generation of cancer-promoting viral mutations during the long-term evolutional process, thus promoting HBV-induced hepatocarcinogenesis.

MicroRNAs (miRNAs), a group of non-coding RNAs with an average length of 22 nt, are powerful post-transcriptional regulators *via* binding to the 3′-untranslated region (3′UTR), coding region or 5′UTR of the target mRNA. Some of miRNAs are deregulated upon HBV infection and function as either oncogenes or tumor suppressors in HBV-induced hepatocarcinogenesis [15]. miRNAs are generally transcribed by RNA polymerase II as long precursors, pre-miRNAs. Pre-miRNA encoding genes are frequently located at fragile sites of human genome where HBV gene is often integrated [16], [17]. The miR-34 family comprises 3 miRNAs: miR-34a, miR-34b, and miR-34c. miR-34b and miR-34c function as tumor suppressors and their expression in several types of cancer were frequently down-regulated *via* epigenetic silencing [18]–[22]. miR-34b and miR-34c are also involved in infection-induced inflammation and regulated by p53, although miR-34 is nonessential in p53 functioning in mice [23]–[25]. miR-196a2 functions like an oncogene and targets several homeobox transcription factor genes or other genes including annexin A1, affecting cell differentiation or oncogenesis [26]–[29]. miR-34b, miR-34c, and miR-196a2 have been related to inflammation and viral infection [24], [30]. miR-34b and miR-34c share a common primary transcript, termed as pri-miR-34b/c. Recently, a potentially functional polymorphism (rs4938723) in the putative promoter region of *pri-miR-34b/c* was found to be associated with an increased risk of HCC in a case-control study [31]. In this pioneering work, however, the odds ratios (ORs) in each subgroup were not adjusted for HBV infection status, the major causal factor of HCC in China, possibly leading to the uncertainties of the associations. *Pre-miR-196a2* SNP rs11614913, which would alter the mature miR-196a2 expression level [29], was also found to be associated with HCC risk [32], [33]. However, the data are confusing because a recent meta-analysis did not support the association of rs11614913 with HCC risk [34]. Furthermore, the effect of miR-34b/c and miR-196a2 on the generation of the HCC-related HBV mutations and the interactions of “gene-gene (locus-locus)” and “gene-viral mutation” on the risk of HBV-HCC remain unknown. We hypothesize that these SNPs might alter the expression and/or function of mature miRNAs and their targeted genes with either potential oncogenic or suppressive function and affect tumor-supporting inflammatory pathways and immune selection of the HBV mutations, thus contributing to HCC risk. Here, we presented data from a large epidemiological study with intact controls (healthy controls and HBV-infected subjects with various clinical conditions), elucidating possible roles of *pri-miR-34b/c* and *pre-miR-196a2* polymorphisms and their interactions with the HCC-related HBV mutations in hepatocarcinogenesis. The findings of this study might be helpful in determining the HBV-infected subjects who are more likely to develop HCC and need specific interventions.

## Methods

### Study Subjects

Healthy controls were selected from those who received annual physical examinations and were proven to be healthy at Health Examination Center, Changhai Hospital of this University from September 2009 to June 2010. They were free of serological HBV parameters including antibody to HBc (anti-HBc) and antibody to HCV (anti-HCV). HBV natural clearance subjects and asymptomatic HBsAg carriers (ASCs) were initially recruited from our HBV-infected subjects cohort established in Yangpu district of Shanghai when we screened for study subjects seropositive for HBsAg in 2010. HBV natural clearance subjects were seronegative for HBsAg, HBV DNA, and anti-HCV, but seropositive for antibodies against HBsAg (anti-HBs) and anti-HBc. No history of HBV vaccination was reported for these subjects. ASCs were seropositive for HBsAg. HBV natural clearance subjects and ASCs were free of clinical liver diseases by examining liver function and serum alpha-fetoprotein and by ultrasonic examination using a Philips iU22 scanner (Philips Medical Systems, Best, The Netherlands) equipped with a 2–4 MHz variable convex probe. The HBV natural clearance subjects and ASCs were revisited during the follow-up in 2011. Those whose diagnoses were the same as the previous examinations were finally involved in this study. Patients with chronic hepatitis B (CHB), HBV-infected patients with liver cirrhosis (LC), and HBV-infected patients with HCC were recruited from Changzheng Hospital, Changhai Hospital, and Eastern Hepatobiliary Surgery Hospital of this university, Southwest hospital, Chongqing, and the 88th hospital in Taian city, Shandong, China, from October 2009 to September 2011. ASC status, CHB, LC, and HCC were diagnosed according to the criteria as previously described [35], [36]. The subjects seropositive for antibodies to HCV or hepatitis delta virus were not included. A total of 1,012 healthy controls, 302 HBV natural clearance subjects, 316 ASCs, 316 patients with CHB, 358 HBV-infected patients with LC, and 1,021 HBV-infected patients with HCC were enrolled in this study. The study subjects were of Han Chinese ancestry.

### Ethics Statement

A written consent was obtained from each study subject. The study protocol conformed to the 1975 Declaration of Helsinki and was approved by the ethics committee of Second Military Medical University.

### Serological Viral Parameters Examination, HBV Genotyping, and Mutation Analysis

HBsAg, anti-HBs, hepatitis B e antigen (HBeAg), anti-HBe, anti-HBc, HBV DNA, anti-HCV, alpha-fetoprotein, liver function parameters including alanine aminotransferase (ALT) were examined as previously described [13], [35], [36]. Antibody to hepatitis delta virus was examined using commercial kits (Wantai Bio-Pharm, Beijing, China). HBV genotyping, PCR amplification of the HBV EnhII/BCP/PC region and the preS region, and viral mutation analysis were carried out as previously reported [35], [37], [38].

### Genotyping of the SNPs

The SNPs were genotyped using fluorescent-probe real-time quantitative PCR in a LightCycler^TM^480 (Roche, Basel, Switzerland). Probes (Minor Groove Binder [MGB]) and primers were commercially designed and synthesized (GeneCore BioTechnologies, Shanghai, China). Sequences of the primers and probes and PCR amplification condition are listed in Table S1. Each reaction mixture contained 0.2 µmol/L of primers and probes, 1–4 ng/µL purified templates in Premix Ex Taq reaction system (Takara, Dalian, China). The genotyping was performed without knowing the participants’ disease status. Two blank controls in each 96-well format were used for quality control, and more than 10% of samples were randomly selected to repeat, yielding a 100% concordance. The success rates of genotyping for all of these SNPs were greater than 99%.

### Statistical Analysis

Hardy-Weinberg equilibrium (HWE) of each SNP in the study population was examined online (http://ihg.gsf.de/ihg/snps.html). The differences in categorical variables were evaluated using the chi-square test. Serum levels of HBV DNA and ALT with skewed distribution were adjusted to normal distribution by transformation into logarithmic function, and then tested by Student’s *t* test or analysis of variance. For the main effects of the SNPs, unconditional logistic regression model was conducted to compute an OR and the 95% confidence interval (95% CI), adjusted for age and gender, respectively. Since HCC is more frequent in men than in women, we stratified our study subjects into gender groups and evaluated the associations of each SNP with HCC risk separately. Contributions of each SNP, its multiplicative interaction with gender, and the multiplicative gene-gene interactions to HCC risk in all study subjects or in the HBV-infected subjects were evaluated using multivariate regression analyses. Contributions of each SNP and its multiplicative interaction with HCC-related HBV mutations to HCC risk in the HBV-infected subjects with HBV sequencing data were also evaluated by multivariate regression analyses, adjusted for covariates including HBV mutations. The HBV mutations in the EnhII/BCP/PC region and those in the preS region were separately evaluated in the multivariate regression analyses because the two HBV fragments were only amplified from partially overlapped fractions of the HBV-infected subjects. All statistical tests were two-sided and performed using SPSS 16.0 for Windows (SPSS, Chicago, IL). A *P* value of <0.05 was considered as statistically significant. *P* values were corrected by the Bonferroni correction for multiple comparisons.

## Results

Age, gender, and HBV infection-related parameters of study subjects are described in Table S2. In brief, HBV natural clearance subjects and healthy controls were older than the HBV-infected subjects including the HCC patients. The HBV-HCC patients were older and had a higher proportion of men compared to the HBV-infected subjects without HCC. In contrast to serum levels of viral load and ALT, the infection with HBV genotype C and HBeAg seroconversion were more frequent in the HBV-HCC patients than in the HBV-infected subjects without HCC. The differences in viral loads were not significant among ASCs, the CHB patients, and the LC patients after Bonferroni correction (cutoff *P* value: 0.010).

### Associations of the *pri-miR-34b/c* and *pre-miR-196a2* Polymorphisms with HCC and Other HBV-related Properties

The genotype distributions of *pri-miR-34b/c* rs4938723 and *pre-miR-196a2* rs11614913 in healthy controls, HBV natural clearance subjects, HCC-free HBV-infected subjects, and HBV-infected subjects with HCC are shown in Table 1. The genotype frequencies for the SNPs in the 4 groups of the study subjects were all conformed to HWE, either in men or in women (*P*>0.05 for each). As compared with the HBV natural clearance subjects *pri-miR-34b/c* rs4938723 CC genotype was significantly associated with an increased risk of HCC after the adjustment for age and gender (AOR = 2.01, 95% CI = 1.16–3.49, *P* = 0.012). *Pri-miR-34b/c* rs4938723 variant genotypes significantly increased HCC risk in women, compared to female healthy controls (AOR = 1.85, 95% CI = 1.20–2.84, *P* = 0.005) or all of female study subjects without HCC (AOR = 1.62, 95% CI = 1.14–2.31, *P* = 0.007) in dominant genetic models (heterozygote/mutational homozygote *vs.* wild homozygote). These *P* values reached the significant level of Bonferroni correction (a cutoff *P* value of 0.013). These effects were not significant in men. *Pre-miR-196a2* rs11614913 was not significantly associated with HCC risk, neither in men nor in women.

**Table 1:** The associations of the miRNA polymorphisms with HCC.

Gender group	Genotype/allele	Healthy controls (%)	HBV natural clearance subjects (%)	HBV-infected subjects withoutHCC (%)	HBV-HCC patients (%)	AOR (95% CI)			
						HBV-HCC patients*vs.* healthy controls	HBV-HCC patients*vs.* HBV clearance subjects	HBV-HCC patients*vs.* HBV-infected subjects withoutHCC	HBV-HCC patients *vs.* all of the controls
*Pri-miR-34b/c*(rs4938723, T >C),combined	TT	456(45.6)	143(47.4)	481(48.9)	451(44.5)	1.00	1.00	1.00	1.00
	TC	424(42.4)	133(44.0)	409(41.6)	444(43.8)	1.06(0.96–1.17)	1.09(0.94–1.26)	1.08(0.97–1.19)	1.05(0.97–1.14)
	CC	119(11.9)	26(7.1)	94(9.6)	118(11.6)	1.14(0.84–1.55)	2.01(1.16–3.49)	1.21(0.88–1.65)	1.20(0.93–1.55)
	C(TC+CC)	543(54.4)	159(52.6)	503(51.1)	562(55.5)	1.13(0.94–1.37)	1.30(0.97–1.73)	1.16(0.97–1.40)	1.13(0.97–1.31)
	HWE *P*	0.18	0.53	0.27	0.30				
rs4938723 in women	TT	125(50.8)	63(47.4)	158(51.1)	64(39.8)	1.00	1.00	1.00	1.00
	TC	93(37.8)	56(42.1)	133(43.0)	77(47.8)	1.38(1.10–1.74)	1.23(0.96–1.59)	1.17(0.95–1.44)	1.25(1.04–1.50)
	CC	28(11.4)	14(10.5)	18(5.8)	20(12.4)	1.72(0.85–3.48)	1.97(0.85–4.55)	2.33(1.14–4.76)	2.03(1.13–3.65)
	C(TC+CC)	121(49.2)	70(52.6)	151(48.9)	97(60.2)	1.85(1.20–2.84)	1.58(0.97–2.55)	1.49(1.00–2.22)	1.62(1.14–2.31)
rs4938723 in men	TT	331(44.0)	80(47.3)	323(47.9)	387(45.4)	1.00	1.00	1.00	1.00
	TC	331(44.0)	77(45.6)	276(40.9)	367(43.1)	1.00(0.90–1.12)	1.01(0.83–1.21)	1.05(0.94–1.17)	1.01(0.93–1.11)
	CC	91(12.1)	12(7.1)	76(11.3)	98(11.5)	1.03(0.73–1.45)	1.95(0.95–3.99)	1.04(0.74–1.47)	1.07(0.81–1.42)
	C(TC+CC)	422(56.0)	89(52.7)	352(52.1)	465(54.6)	1.01(0.82–1.25)	1.13(0.79–1.63)	1.09(0.88–1.34)	1.04(0.88–1.23)
*Pre-miR-196a2*(rs11614913, T >C),combined	TT	304(30.1)	85(28.1)	289(29.4)	305(30.0)	1.00	1.00	1.00	1.00
	TC	485(48.1)	158(52.3)	467(47.5)	505(49.7)	1.02(0.92–1.14)	0.96(0.81–1.13)	1.00(0.90–1.11)	1.00(0.91–1.09)
	CC	220(21.8)	59(19.5)	227(23.1)	207(20.4)	1.00(0.76–1.30)	1.07(0.70–1.62)	0.79(0.61–1.03)	0.91(0.73–1.13)
	C(TC+CC)	705(69.9)	217(71.9)	694(70.6)	712(70.0)	1.03(0.84–1.27)	0.95(0.70–1.31)	0.93(0.76–1.14)	0.97(0.82–1.14)
	HWE *P*	0.31	0.34	0.19	0.94				
rs11614913 in women	TT	79(31.9)	36(27.1)	96(31.2)	52(32.5)	1.00	1.00	1.00	1.00
	TC	114(46.0)	66(49.6)	135(43.8)	78(48.8)	0.97(0.76–1.23)	0.91(0.69–1.20)	1.01(0.81–1.27)	0.98(0.81–1.20)
	CC	55(22.2)	31(23.3)	77(25.0)	30(18.8)	0.75(0.41–1.36)	0.67(0.34–1.32)	0.65(0.37–1.15)	0.70(0.42–1.15)
	C(TC+CC)	169(68.1)	97(72.9)	212(68.8)	108(67.5)	0.87(0.55–1.38)	0.78(0.46–1.30)	0.89(0.58–1.36)	0.87(0.60–1.27)
rs11614913 in men	TT	225(17.3)	49(29.0)	193(28.6)	253(29.5)	1.00	1.00	1.00	1.00
	TC	371(28.6)	92(54.4)	332(49.2)	427(49.8)	1.04(0.92–1.18)	0.99(0.80–1.22)	0.99(0.88–1.12)	1.00(0.91–1.10)
	CC	165(12.7)	28(16.6)	150(22.2)	177(20.7)	1.07(0.80–1.44)	1.47(0.85–2.55)	0.83(0.62–1.12)	0.97(0.76–1.23)
	C(TC+CC)	536(41.3)	120(71.0)	482(71.4)	604(70.5)	1.08(0.86–1.36)	1.10(0.74–1.62)	0.94(0.74–1.18)	0.99(0.83–1.19)

AOR, adjusted odds ratio (adjusted for age and gender in the total subjects; adjusted for age after stratification by gender); CI, confidence interval; HBV, hepatitis B virus; HCC, hepatocellular carcinoma; HWE, Hardy-Weinberg equilibrium.

None of the SNPs were associated with HBV natural clearance, HCC-free chronic HBV infection, abnormal ALT (<40 *vs.* ≥40 U/L), and viral load (<10^4^ *vs.* ≥10^4^ copies/mL), adjusted for age and gender or for age after stratification by gender. However, *pri-miR-34b/c* rs4938723 CC genotype was inversely associated with the risk of LC compared to ASCs plus the CHB patients, adjusted for age and gender (AOR = 0.60, 95% CI = 0.36–0.99); this effect was only found in male study subjects in dominant genetic model (AOR = 0.68, 95% CI = 0.49–0.93) (Table S3).

### Contributions of the Multiplicative Interaction of the SNPs with Gender and Gene-gene Interaction to the Risk of HCC

Multivariate regression analyses were firstly performed to determine the contribution of the multiplicative interaction of each SNP with gender (men *vs.* women) to HCC risk in the whole study subjects or in the HBV-infected subjects, adjusted for age. It was found that *pri-miR-34b/c* rs4938723 in dominant genetic model was significantly associated with an increased risk of HCC, with an AOR of 1.62 (95% CI = 1.14–2.31), whereas its multiplicative interaction with men (*vs.* women) were inversely associated with HCC risk, with an AOR of 0.64 (95% CI = 0.43–0.94), in the whole study subjects. *Pri-miR-34b/c* rs4938723 in dominant genetic model tended to be associated with an increased risk of HCC in the HBV-infected subjects (AOR = 1.49, 95% CI = 1.00–2.23) (Table 2). *Pre-miR-196a2* rs11614913 and its multiplicative interaction with gender were not significantly associated with HCC risk, neither in the whole study subjects nor in the HBV-infected subjects (data not shown).

**Table 2:** Contribution of multiplicative interactions *pri-miR-34b/c* (rs4938723, T >C) with gender to HCC risk in multivariate regression analyses.

Variables	AOR (95% CI)	*P* value
**HBV-HCC patients (n = 1,013) ** ***vs.*** ** all study subjects without HCC (n = 2,110)**		
Age (year)	1.00(0.99–1.00)	0.138
Gender (men *vs.* women)	2.82(2.10–3.80)	<0.001
rs4938723 (TC+CC *vs.* TT)	1.62(1.14–2.31)	0.007
rs4938723 (TC+CC *vs.* TT) × gender (men *vs.* women)	0.64(0.43–0.94)	0.023
**HBV-HCC patients (n = 1,013) ** ***vs.*** ** HBV-infected subjects without HCC (n = 969)**		
Age (year)	1.05(1.04–1.05)	<0.001
Gender (men *vs.* women)	2.96(2.11–4.14)	<0.001
rs4938723 (TC+CC *vs.* TT)	1.49(1.00–2.23)	0.050
rs4938723 (TC+CC *vs.* TT) × gender (men *vs.* women)	0.73(0.46–1.14)	0.168

AOR, adjusted odds ratios; CI, confidence interval; HBV, hepatitis B virus; HCC, hepatocellular carcinoma.

In the multivariate regression model, the multiplicative interaction of rs4938723 with rs11614913 was not significantly associated with HCC risk in the whole study subjects. However, the interaction of rs4938723 TC genotype with rs11614913 CC genotype was significantly associated with an increased risk of HCC in women, with an AOR of 2.21 (95% CI = 1.24–3.94, *P* = 0.007) (Table S4).

### Associations of the SNPs with the Frequencies of the HCC-related HBV Mutations

HBV EnhII/BCP/PC region was successfully sequenced from 57.68% of the HBsAg-positive subjects (79.75% of ASCs, 54.43% of the CHB patients, 62.57% of the LC patients, and 50.15% of the HCC patients); the preS region was sequenced from 47.14% of the HBsAg-positive subjects (41.14% of ASCs, 51.90% of the CHB patients, 54.19% of the LC patients, and 45.05% of the HCC patients). Of the HBV-infected subjects, 741 (36.85%) had the HBV mutation data in the two regions. The HBV mutations including T1674C/G, A1762T/G1764A, T1753V, C1653T, and G1896A in the EnhII/BCP/PC as well as preS deletion, preS1 start codon mutation, preS2 start codon mutation, C2875A, A3120G/T, C7A, and C76A in the preS region were significantly associated with an increased risk of HCC, while C1730G and C1799G were inversely associated with HCC risk [39]. The associations of the SNPs with the frequencies of all the HCC-related HBV mutations were assessed using the data of the HBV-infected subjects including the HCC patients. It was found that *pri-miR-34b/c* rs4938723 CC genotype was significantly associated with increased frequency of T1674C/G, while *pre-miR-196a2* rs11614913 TC genotype was significantly associated with increased frequency of G1896A (Table 3).

**Table 3:** The associations of the polymorphism with the HCC-related HBV mutations using the data of all HBV-infected subjects.

SNP	Genotype/allele	T1674C/G				G1896A			
		T(%)	C/G(%)	AOR (95% CI)	*P* value	G (%)	A (%)	AOR (95% CI)	*P* value
*Pri-miR-34b/c*(rs4938723, T >C)	TT	358(47.3)	102(40.8)	1.00		282(44.2)	191(46.9)	1.00	
	TC	322(42.5)	108(43.2)	1.09(0.93–1.28)	0.269	285(44.7)	167(41.0)	0.93(0.81–1.06)	0.278
	CC	77(10.2)	40(16.0)	1.77(1.13–2.76)	0.012	71(11.1)	49(12.0)	0.99(0.65–1.50)	0.959
	C(TC+CC)	399(52.7)	148(59.2)	1.31(0.98–1.75)	0.073	356(55.8)	216(53.1)	0.89(0.69–1.14)	0.358
*Pre-miR-196a2*(rs11614913, T >C)	TT	205(27.0)	75(29.9)	1.00		197(30.8)	109(26.8)	1.00	
	TC	379(50.0)	119(47.4)	0.93(0.79–1.10)	0.403	291(45.5)	222(54.5)	1.17(1.01–1.36)	0.035
	CC	174(23.0)	57(22.7)	0.87(0.58–1.30)	0.487	152(23.8)	76(18.7)	0.84(0.58–1.22)	0.353
	C(TC+CC)	553(73.0)	176(70.1)	0.87(0.63–1.19)	0.370	443(69.2)	298(73.2)	1.19(0.90–1.58)	0.216

AOR, odds ratios adjusted for age and gender in the total subjects; CI, confidence interval; HBV, hepatitis B virus.

### Contributions of the Interactions of the SNPs with the HBV Mutations to HCC Risk

Contribution of each SNP and its multiplicative interaction with the HCC-related HBV mutations to HCC risk were assessed using multivariate regression analyses, adjusted for covariates including the HBV mutations. The contributions of the SNPs and their multiplicative interactions with HBV mutations in the EnhII/BCP/PC region or in the preS region were calculated by adding each SNP and the interaction term to the same multivariate regression model. In the study subjects with the data of HBV mutations in the EnhII/BCP/PC region, *pri-miR-34b/c* rs4938723 in dominant genetic model was significantly associated with an increased risk of HCC whereas its interaction with C1730G, a HBV mutation inversely associated with HCC risk, was significantly associated with a reduced risk of HCC; the interaction of *pre-miR-196a2* rs11614913 TC genotype with G1896A was significantly associated with an increased risk of HCC (Table 4). In those with the data of HBV mutations in the preS region, *pri-miR-34b/c* rs4938723 in dominant genetic model was significantly associated with an increased risk of HCC whereas its interactions with viral mutations were not significantly associated with HCC risk; the interaction of *pre-miR-196a2* rs11614913 TC genotype with A3120G/T was significantly associated with a reduced risk of HCC, although A3120G/T was a risk factor of HCC (Table 5).

**Table 4:** Contributions of *pri-miR-34b/c* and *pre-miR-196a2* polymorphisms and their interactions with the HBV mutations in the EnhII/BCP/PC region to the risk of HCC in multivariate regression analyses.

Variables	AOR (95% CI)	*P* value
***Pri-miR-34b/c*** ** rs4938723 (T >C) equation** *		
Age (year)	1.04(1.02–1.06)	<0.001
Gender (men *vs.* women)	1.99(1.28–3.09)	0.002
Viral load (≥10^4^ *vs.* <10^4^ copies/mL)	1.08(0.74–1.58)	0.677
HBV genotype (C *vs.* B)	0.77(0.44–1.36)	0.371
C1730G	1.88(0.94–3.74)	0.075
T1674C/G	2.11(1.33–3.34)	0.001
T1753V	1.89(1.21–2.94)	0.005
G1896A	1.74(1.20–2.53)	0.004
rs4938723 (TC+CC *vs.* TT)	1.62(1.05–2.49)	0.028
rs4938723 (TC+CC *vs.* TT) **×**C1730G	0.34(0.15–0.81)	0.014
***Pre-miR-196a2*** ** rs11614913 (T >C) equation** †		
Age (year)	1.04(1.02–1.06)	<0.001
Gender (men *vs.* women)	2.63(1.57–4.38)	<0.001
Viral load (≥10^4^ *vs.* <10^4^ copies/mL)	1.35(0.88–2.07)	0.165
HBV genotype (C *vs.* B)	0.83(0.44–1.55)	0.556
C1730G	1.32(0.70–2.48)	0.394
T1674C/G	2.01(1.20–3.37)	0.008
T1753V	2.01(1.20–3.37)	0.008
G1896A	0.95(0.47–1.92)	0.880
rs11614913 (TC *vs.* TT)	0.82(0.61–1.09)	0.173
rs11614913 (TC *vs.* TT)×G1896A	1.63(1.05–2.54)	0.029

AOR, adjusted odds ratio; CI, confidence interval; EnhII/BCP/PC, enhancer II/basal core promoter/precore; HBV, hepatitis B virus; HCC, hepatocellular carcinoma.

* HBV-HCC patients (effective number [n] = 340) *vs*. HBV-infected subjects without HCC (n = 224).

† HBV-HCC patients (n = 276) *vs*. HBV-infected subjects without HCC (n = 171).

**Table 5:** Contributions of miRNA polymorphisms and their interactions with the HBV mutations in the preS region to HCC risk in multivariate regression analyses.

Variables	AOR (95% CI)	*P* value
***Pri-miR-34b/c*** ** rs4938723 (T>C) equation** *		
Age (year)	1.05(1.04–1.07)	<0.001
Gender (men *vs.* women)	3.28(2.04–5.26)	<0.001
Viral load (≥10^4^ *vs.* <10^4^ copies/mL)	0.40(0.27–0.60)	<0.001
HBV genotype (C *vs.* B)	4.07(1.85–8.94)	0.001
A3120G/T	2.29(1.19–4.40)	0.013
C2875A	1.50(0.83–2.69)	0.180
preS1 start codon mutation	2.46(0.96––6.31)	0.061
C76A	2.28(1.00–5.20)	0.051
preS2 start codon mutation	2.36(1.40–3.99)	0.001
rs4938723 (TC+CC *vs.* TT)	1.55(1.02–2.38)	0.043
rs4938723 (TC+CC *vs.* TT) × preS1 start codon mutation	0.93(0.26–3.39)	0.912
***Pre-miR-196a2*** **rs11614913 (T>C) equation** †		
Age (year)	1.06(1.04–1.08)	<0.001
Gender (men *vs.* women)	3.80(2.24–6.44)	<0.001
Viral load (≥10^4^ *vs.* <10^4^ copies/mL)	0.39(0.25–0.61)	<0.001
HBV genotype (C *vs.* B)	3.64(1.54–8.64)	0.003
A3120G/T	5.62(1.91–16.51)	0.002
C2875A	1.45(0.75–2.79)	0.266
preS1 start codon mutation	2.17(1.05–4.51)	0.038
C76A	2.20(0.91–5.35)	0.082
preS2 start codon mutation	2.30(1.31–4.06)	0.004
rs11614913 (TC *vs.* TT)	1.15(0.88–1.50)	0.301
rs11614913 (TC *vs.* TT)×A3120G/T	0.53(0.30–0.92)	0.024

AOR, adjusted odds ratio; CI, confidence interval; HBV, hepatitis B virus; HCC, hepatocellular carcinoma; miRNA, microRNA.

* HBV-HCC patients (effective number [n] = 311) *vs.* HBV-infected subjects without HCC (n = 210).

† HBV-HCC patients (n = 255) *vs.* HBV-infected subjects without HCC (n = 170).

## Discussion

In this study, we found that *pri-miR-34b/c* rs4938723 variant genotypes in dominant genetic model significantly increased HCC risk in women as compared with the female subjects without HCC. Furthermore, the interaction of rs4938723 in dominant genetic model with female gender was significantly associated with HCC risk. HCC is more common in men than in women, especially in the HBV-infected population [2], [3]. This gender disparity might be caused by sex hormone signaling, genetic predisposition such as polysomy of chromosome 7, and increased exposure to environmental risk factors in men [40]–[42]. Heavy alcohol consumption has been reported to increase HCC risk in male HBsAg carriers [43]. In China, heavy alcohol consumption is more common in men than in women. Thus, the rs4938723 effect on genetic susceptibility to HBV-HCC might be overwhelmed by strong effects of these risk factors in men. The effect in women might reflect a less biased association of rs4938723 with HBV-HCC. rs4938723 (chr.111382565) is located at the putative promoter region of *pre-miR-34b/c*. Since miR-34b/c has tumor suppression function, the mutation genotypes (TC and CC) of rs4938723 should be associated with the down-regulation of miR-34b/c in liver. To evaluate if rs4938723 with a base T-to-C change might alter the binding sites of transcriptional factors, we carried out an *in silico* assay by downloading a 50 bp genomic sequence covering rs4938723 (chr.111382539-chr.111382588) to predict transcriptional factors binding sites online (http://www.cbrc.jp/research/db/TFSEARCH.html). It was found that the T-to-C change at rs4938723 introduced binding sites of GATA-1, GATA-2, GATA-3, GATA-X, and AP-4 in the 50bp genomic sequence (Figure S1). GATA transcriptional factors can be regulated by gonadotropin and other sexual hormones and control gonadal development and sex differentiation in mammals [44]. GATA-2 plays an important role in activating androgen receptor signaling in prostate cancer [45]. GATA-3, in association with estrogen receptor, can regulate genes critical to the hormone-responsive breast cancer phenotype and play a crucial role for the response of estrogen receptor alpha-positive breast cancers to estradiol [46], [47]. Thus, we speculate that sexual hormones and their receptors might affect the function of GATA transcriptional factors binding to the putative promoter sequence with the T-to-C change at rs4938723, and regulate the expression of *pri-miR-34b/c*. This speculation needs to be confirmed by biological assays in future studies.

Infection and nonresolving inflammation contribute to about a quarter of all cancer cases. Inflammation modulates the expressions of miRNAs that influence the production of tumor-related proteins [24], [48]. miRNAs possibly link nonresolving inflammation and cancers. From the data reported here (Table 1 and Table S3) and elsewhere [31], *pri-miR-34b/c* rs4938723 might not be directly involved in active inflammation in liver. The rs4938723 effect on HCC risk might be related to the tumor suppression function of miR-34b/c. If so, the expression of miR-34b/c in liver with rs4938723 variant genotypes tends to be down-regulated. In the HBV-infected subjects with the data of HBV mutations, however, *pri-miR-34b/c* rs4938723 CC genotype was significantly associated with increased frequencies of HCC-related HBV mutation T1674C/G (Table 3). miR-34b/c might be involved in immune selection of T1674C/G and this immune selection might be independent of exacerbation during chronic HBV infection.

The mutated genotype (TC and CC) of rs11614913, which is located at 3′ passenger strand mature miR-196a2, has been associated with increased risk of digestive system cancers [49]. Since the expression level of miR-196a2 correlates with the wild-type genotype (TT) of rs11614913 [28], [29], the mutated genotypes might be associated with low expression of miR-196a2. In this study, we did not find a significant association of rs11614913 genotype with the genetic susceptibility to HCC, which is in accordance with the data of the meta-analysis [34]. In the present study, the controls covered HBV-infected and -uninfected subjects with statistically acceptable sample size. These results should be lack of bias introduced by incomplete controls. In this study, we found that the interaction of rs11614913 CC genotype with rs4938723 TC genotype significantly increased the risk of HCC in women, suggesting that miR-196a2, miR-34b/c, and their targeting pathways might have intrinsic cross-talks in hepatocarcinogenesis in women.

In a multivariate regression equation, *pri-miR-34b/c* rs4938723 variant genotypes in dominant genetic model was significantly associated with an increased risk of HCC; however, its interaction with C1730G, a HBV mutation inversely associated with HCC risk, was significantly associated with a reduced risk of HCC (Table 4). Thus, the rs4938723 effect on HCC risk can be strongly affected by the HBV mutations. *Pre-miR-196a2* rs11614913 TC genotype was significantly associated with increased frequencies of HCC-related HBV mutation G1896A (Table 3), furthermore, the interaction of this genotype with G1896A significantly increased HCC risk (Table 4); whereas the interaction of this genotype with A3120G/T, a strong HCC-related HBV mutation, significantly reduced the risk of HCC (Table 5). Thus, *pre-miR-196a2* rs11614913 TC genotype has a synergistic effect with G1896A and an antagonistic effect with A3120G/T on HCC risk. The effect of *pre-miR-196a2* rs11614913 on HCC risk might be neutralized in the patients with both of G1896A and A3120G/T. Although the mechanisms by which rs4938723 or rs11614913 has an antagonistic or synergistic effect with the HBV mutations on HCC occurrence need to be further elucidated, our data reflect the complexity of the host gene-virus and gene-gene interactions in HBV-induced hepatocarcinogenesis and also present a direction of exploring genetic risk factors of cancers whose occurrences are strongly influenced by environmental factors.

Our study has limitations. First, we failed to amplify the two HBV fragments from all of HBV-infected population, resulting in a possible preponderance of missing data. Failure to amplify the HBV region might be due to low viral concentration in sera or HBV mutations in the primer binding sites. Second, other environmental exposures such as alcohol consumption and family history of cancer were incomplete and thus not included in the analyses. Third, healthy controls were selected from one of the hospitals from which cases were recruited. This may cause potential selection bias. Fourth, the study design is cross-sectional in nature. Future prospective studies are needed to confirm the synergistic or antagonistic effect of the SNPs with the HBV mutations in hepatocarcinogenesis and define the HBV-infected subjects who are more likely to develop HCC and need specific interventions.

In conclusion, our study suggested that *pri-miR-34b/c* rs4938723 was associated with a significant increased risk of HCC, especially in women. Although *pre-miR-196a2* rs11614913 was not statistically associated with HCC risk, it may enhance the effect of *pri-miR-34b/c* rs4938723 in women. rs4938723 CC genotype and rs11614913 TC genotype might predispose the host to immune selection of T1674C/G, and G1896A, respectively. The rs4938723 effect on HCC risk can be seriously affected by the HBV mutations. In light of our results, well-designed prospective studies with ethnically diverse HBV-infected populations and functional studies are warranted to elucidate the interaction of the SNPs with the HBV mutations in hepatocarcinogenesis.

## Supporting Information

Figure S1 **Schematic diagram of altered binding sites of transcription factors in the putative promoter region of** ***pri-miR-34b/c*** **due to the T-to-C change at rs4938723.**
(TIF)

Table S1 **The primers, probes, and PCR program for genotyping the polymorphisms.**
(DOC)

Table S2 **Age, gender, and HBV infection-related parameters of the subjects enrolled in this study.**
(DOC)

Table S3 **The associations of the polymorphisms with HCC-free chronic HBV infection, LC, abnormal ALT and high viral load.**
(DOC)

Table S4 **Association of multiplicative interaction of** ***pri-miR-34b/c*** **rs4938723 and** ***pre-miR-196a2*** **rs11614913 with HCC risk in multivariate regression analyses.**
(DOC)
