# Supplementary figures and images for: Associations of pri-miR-34b/c and pre-miR-196a2 Polymorphisms and Their Multiplicative Interactions with Hepatitis B Virus Mutations with Hepatocellular Carcinoma Risk
Source: PLoS One. 2013 Mar 13;8(3):e58564. doi: 10.1371/journal.pone.0058564 (PMC3596299; doi:10.1371/journal.pone.0058564)

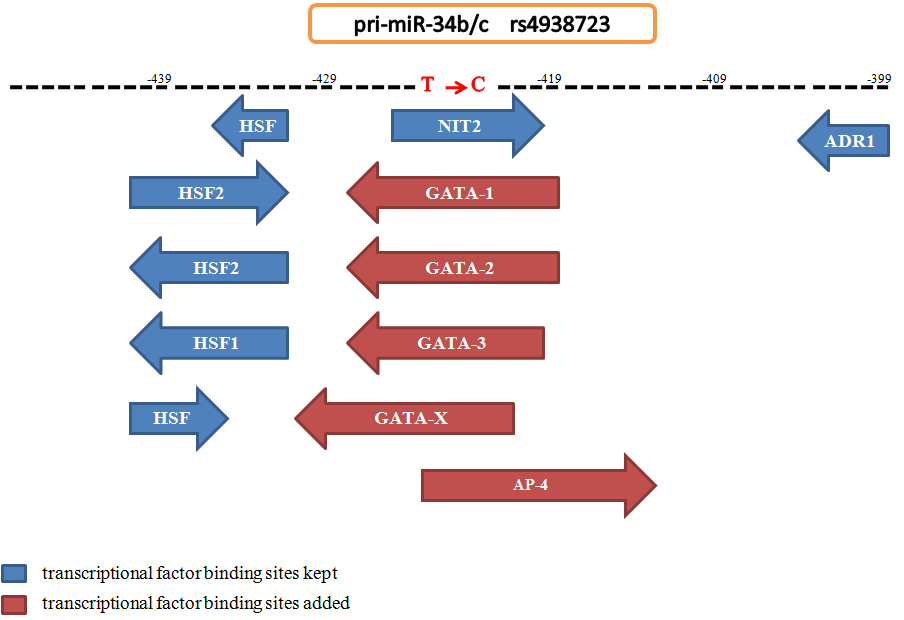

Supplement: Figure S1 — Schematic diagram of altered binding sites of transcription factors in the putative promoter region of pri-miR-34b/c due to the T-to-C change at rs4938723. (TIF) [file pone.0058564.s001.tif]
