# Supplementary material for: Associations of pri-miR-34b/c and pre-miR-196a2 Polymorphisms and Their Multiplicative Interactions with Hepatitis B Virus Mutations with Hepatocellular Carcinoma Risk
Source: PLoS One. 2013 Mar 13;8(3):e58564. doi: 10.1371/journal.pone.0058564 (PMC3596299; doi:10.1371/journal.pone.0058564)
Supplement: Table S1 — The primers, probes, and PCR program for genotyping the polymorphisms. (DOC) [file pone.0058564.s002.doc]

**Table S1.** The primers, probes, and PCR program for genotyping the polymorphisms

| **Polymorphisms** | **Names** | **Sequence (5’-3’)** | **Alleles** | **PCR program** |
| --- | --- | --- | --- | --- |
| *pri-miR-34b/c* rs4938723 | Forward | TGT ACT CGT GCA TCA AGG ATC TAC TC | T/C | step 1 95ºC for 10s  step 2 45 cycles of 95ºC for 10s and 60ºC for 30s  step 3 40ºC for 1s |
|  | Reverse | CAG AAA TAG AAG GGA GGT CCT CAA T |  |  |
|  | Probe-P1 | FAM -TTG ACC TAT TAC AGC TCT -MGB |  |  |
|  | Probe-P2 | HEX -TGA CCT ATC ACA GCT C -MGB |  |  |
| *Pre-miR-196a2*  rs11614913 | Forward | TAG GAG TGG GAG AGG TGG GTT | T/C |  |
|  | Reverse | AGG TAG TTT CAT GTT GTT GGG ATT G |  |  |
|  | Probe-P1 | FAM -TAA CTC AGT CAG TTT CT - MGB |  |  |
|  | Probe-P2 | HEX -TAA CTC AGC CAG TTT C- MGB |  |  |
