# Supplementary material for: Associations of pri-miR-34b/c and pre-miR-196a2 Polymorphisms and Their Multiplicative Interactions with Hepatitis B Virus Mutations with Hepatocellular Carcinoma Risk
Source: PLoS One. 2013 Mar 13;8(3):e58564. doi: 10.1371/journal.pone.0058564 (PMC3596299; doi:10.1371/journal.pone.0058564)
Supplement: Table S2 — Age, gender, and HBV infection-related parameters of the subjects enrolled in this study. (DOC) [file pone.0058564.s003.doc]

**Table S2.** Age, gender, and HBV infection-related parameters of the subjects enrolled in this study

| **Characteristics** | **Healthy controls (n=1012)** | **HBV natural clearance subjects (n=302)** | **HBV-infected subjects without HCC** | | | **HBV-infected subjects with HCC (n=1021)** | ***P* value** |
| --- | --- | --- | --- | --- | --- | --- | --- |
|  |  |  | **ASC (n=316)** | **CHB (n=316)** | **LC (n=358)** |  |  |
| Male (%) | 763(75.40) | 169(55.96) | 186(58.86) | 230(72.78) | 264(73.74) | 864(84.13) | < 0.001^*,†,§,¶^ 0.001^‡^ |
| Age (mean ± SD) | 59.56±15.10 | 58.40±11.72 | 45.08±10.61 | 44.18±14.51 | 50.68±11.34 | 52.92±11.17 | < 0.001^*,†,‡,§,¶^ |
| HBV genotype (%) |  |  |  |  |  |  |  |
| B | ND | ND | 97(34.28) | 52(25.00) | 56(22.86) | 107(16.39) | < 0.001^†^ |
| C | ND | ND | 186(65.72) | 156(75.00) | 189(77.14) | 546(83.61) |  |
| HBeAg (%) |  |  |  |  |  |  |  |
| Positive | ND | ND | 130(41.14) | 132(45.36) | 107(35.55) | 241(25.08) | < 0.001^†^ |
| Negative | ND | ND | 186(58.86) | 159(54.64) | 194(64.45) | 720(74.92) |  |
| HBV DNA (log_10_ copies/mL) | ND | ND | 3.88±1.80 | 4.43±1.67 | 4.13±1.37 | 3.83±1.18 | < 0.001^†^ |
| ALT (log_10_ U/L)) | ND | ND | 1.36±0.21 | 1.97±0.54 | 1.75±0.44 | 1.66±0.35 | < 0.001^†^ |

ASC, asymptomatic hepatitis B surface antigen carrier; CHB, chronic hepatitis B; LC, liver cirrhosis; HCC, hepatocellular carcinoma; HBV, hepatitis B virus; HBeAg, hepatitis B e antigen; ALT, alanine aminotransferase; ND, no data.

^*^ Between HBV-infected subjects with HCC and healthy controls.

† Between HBV-infected subjects with HCC and HBV-infected subjects without HCC.

‡ Between HBV-infected subjects without HCC and healthy controls.

§ Between HBV-infected subjects with HCC and HBV natural clearances.

¶ Between HBV-infected subjects without HCC and HBV natural clearances.

For multiple comparisons, *P* value was corrected by the Bonferroni correction (*P*=0.010).
