# Supplementary material for: Associations of pri-miR-34b/c and pre-miR-196a2 Polymorphisms and Their Multiplicative Interactions with Hepatitis B Virus Mutations with Hepatocellular Carcinoma Risk
Source: PLoS One. 2013 Mar 13;8(3):e58564. doi: 10.1371/journal.pone.0058564 (PMC3596299; doi:10.1371/journal.pone.0058564)
Supplement: Table S3 — The associations of the polymorphisms with HCC-free chronic HBV infection, LC, abnormal ALT and high viral load. (DOC) [file pone.0058564.s004.doc]

**Table S3.** The associations of the polymorphisms with HCC-free chronic HBV infection, LC, abnormal ALT and high viral load

| **SNPs** | **Genotypes/Alleles** | **AOR (95% CI)** | | | | |
| --- | --- | --- | --- | --- | --- | --- |
|  |  | **LC *vs.* ASC+CHB** | **ALT ≥40 *vs.* <40 U/L ^*^** | **HBV DNA ≥10^4^ *vs.* <10^4^copies/mL^*^** | **HBV chronic infection *vs.* HBV natural clearance ^*^** | **HCC-free HBV-infected subjects *vs.* healthy controls ^†^** |
| *Pri-miR-34b/c* ( rs4938723, T>C), combined | TT | 1.00 | 1.00 | 1.00 | 1.00 | 1.00 |
|  | TC | 0.93(0.81-1.07) | 0.93(0.83-1.04) | 1.00(0.89-1.12) | 1.03(0.89-1.18) | 0.99(0.89-1.10) |
|  | CC | **0.60(0.36-0.99)** | 0.81(0.57-1.16) | 0.89(0.63-1.27) | 1.52(0.92-2.53) | 0.84(0.60-1.18) |
|  | C(TC+CC) | 0.81(0.62-1.07) | 0.85(0.68-1.05) | 0.97(0.78-1.20) | 1.13(0.86-1.47) | 0.95(0.77-1.16) |
| rs4938723 in women | TT | 1.00 | 1.00 | 1.00 | 1.00 | 1.00 |
|  | TC | 1.21(0.92-1.58) | 0.97(0.76-1.23) | 1.07(0.84-1.37) | 1.10(0.89-1.36) | 1.10(0.89-1.35) |
|  | CC | 0.62(0.19-2.07) | 0.68(0.28-1.68) | 0.92(0.37-2.32) | 1.19(0.56-2.53) | 0.64(0.31-1.31) |
|  | C(TC+CC) | 1.31(0.78-2.21) | 0.90(0.56-1.43) | 1.12(0.69-1.80) | 1.20(0.79-1.81) | 1.07(0.72-1.58) |
| rs4938723 in men | TT | 1.00 | 1.00 | 1.00 | 1.00 | 1.00 |
|  | TC | 0.84(0.71-0.99) | 0.91(0.80-1.04) | 0.97(0.85-1.10) | 0.98(0.81-1.17) | 0.95(0.84-1.08) |
|  | CC | 0.59(0.34-1.01) | 0.83(0.56-1.22) | 0.86(0.59-1.26) | 1.81(0.90-3.65) | 0.91(0.62-1.34) |
|  | C(TC+CC) | **0.68(0.49-0.93)** | 0.83(0.65-1.06) | 0.92(0.73-1.17) | 1.06(0.75-1.51) | 0.91(0.72-1.15) |
| *Pre-miR-196a2* (rs11614913, T>C), combined | TT | 1.00 | 1.00 | 1.00 | 1.00 | 1.00 |
|  | TC | 1.15(0.98-1.34) | 0.97(0.86-1.10) | 1.02(0.90-1.15) | 0.96(0.82-1.12) | 1.04(0.93-1.17) |
|  | CC | 0.74(0.50-1.09) | 0.92(0.68-1.25) | 0.80(0.59-1.10) | 1.19(0.81-1.75) | 1.21(0.91-1.60) |
|  | C(TC+CC) | 1.11(0.82-1.49) | 0.94(0.74-1.18) | 0.96(0.76-1.21) | 0.99(0.74-1.33) | 1.13(0.90-1.40) |
| rs11614913 in women | TT | 1.00 | 1.00 | 1.00 | 1.00 | 1.00 |
|  | TC | 1.06(0.78-1.44) | 0.94(0.71-1.23) | 1.11(0.84-1.46) | 0.91(0.71-1.16) | 0.97(0.77-1.22) |
|  | CC | 0.70(0.34-1.43) | 0.60(0.31-1.17) | 0.77(0.39-1.54) | 0.86(0.49-1.52) | 1.13(0.66-1.92) |
|  | C(TC+CC) | 0.95(0.54-1.67) | 0.78(0.47-1.31) | 1.08(0.64-1.81) | 0.84(0.53-1.32) | 1.00(0.65-1.53) |
| rs11614913 in men | TT | 1.00 | 1.00 | 1.00 | 1.00 | 1.00 |
|  | TC | 1.17(0.97-1.41) | 0.98(0.85-1.13) | 0.99(0.86-1.14) | 0.99(0.81-1.21) | 1.07(0.93-1.23) |
|  | CC | 0.76(0.47-1.21) | 1.03(0.73-1.46) | 0.82(0.58-1.16) | 1.58(0.92-2.70) | 1.24(0.89-1.72) |
|  | C(TC+CC) | 1.16(0.82-1.66) | 0.98(0.76-1.27) | 0.93(0.71-1.21) | 1.12(0.77-1.65) | 1.18(0.91-1.53) |

AOR, adjusted odds ratio (adjusted for age and gender in the total subjects; adjusted for age after stratification by gender); ASC, asymptomatic hepatitis B surface antigen carrier; CHB, chronic hepatitis B; CI, confidence interval; HBV, hepatitis B virus; HCC, hepatocellular carcinoma; ALT, alanine aminotransferase; LC, liver cirrhosis; SNP, single nucleotide polymorphism.

^*^All HBV-infected subjects including the HCC patients.

**^†^** HCC-free HBV-infected subjects: ASCs, CHB patients, and LC patients.
