# Supplementary material for: Associations of pri-miR-34b/c and pre-miR-196a2 Polymorphisms and Their Multiplicative Interactions with Hepatitis B Virus Mutations with Hepatocellular Carcinoma Risk
Source: PLoS One. 2013 Mar 13;8(3):e58564. doi: 10.1371/journal.pone.0058564 (PMC3596299; doi:10.1371/journal.pone.0058564)
Supplement: Table S4 — Association of multiplicative interaction of pri-miR-34b/c rs4938723 and pre-miR-196a2 rs11614913 with HCC risk in multivariate regression analyses. (DOC) [file pone.0058564.s005.doc]

**Table S4**. Association of multiplicative interaction of *pri-miR-34b/c* rs4938723 and *pre-miR-196a2* rs11614913 with HCC risk in multivariate regression analyses

| **Variables** | **AOR (95% CI)** | ***P* value** |
| --- | --- | --- |
| Combined^*^ | | |
| Age (year) | 1.00(0.99-1.01) | 0.877 |
| Gender (men *vs.* women) | 2.44(1.83-3.26) | < 0.001 |
| rs4938723 (TC *vs.* TT) | 1.00(0.86-1.16) | 0.998 |
| rs11614913 (CC *vs.* TT) | 0.92(0.67-1.27) | 0.623 |
| rs4938723 (TC *vs.* TT) **×**rs11614913 (CC *vs.* TT) | 1.00(0.80-1.26) | 0.979 |
| Men^†^ |  |  |
| Age (year) | 1.00(0.99-1.01) | 0.370 |
| rs4938723 (TC *vs.* TT) | 1.00(0.85-1.18) | 0.981 |
| rs11614913 (CC *vs.* TT) | 1.14(0.80-1.62) | 0.472 |
| rs4938723 (TC *vs.* TT) **×**rs11614913 (CC *vs.* TT) | 0.85(0.66-1.10) | 0.225 |
| Women^‡^ |  |  |
| Age (year) | 1.02(1.00-1.04) | 0.062 |
| rs4938723 (TC *vs.* TT) | 1.01(0.72-1.41) | 0.967 |
| rs11614913 (CC *vs.* TT) | 0.31(0.13-0.76) | 0.011 |
| rs4938723 (TC *vs.* TT) **×**rs11614913 (CC *vs.* TT) | 2.21(1.24-3.94) | 0.007 |

AOR, adjusted odds ratio; CI, confidence interval; HCC, hepatocellular carcinoma.

^*^HBV-HCC patients (effective number [n]=452) *vs.* all study subjects without HCC (n=989).

^†^ HBV-HCC patients (n=382) *vs.* all study subjects without HCC (n=683).

^‡^ HBV-HCC patients (n=70) *vs.* all study subjects without HCC (n=306).
